# Maternal Deworming Research Study (MADRES) protocol: a double-blind, placebo-controlled randomised trial to determine the effectiveness of deworming in the immediate postpartum period

**DOI:** 10.1136/bmjopen-2015-008560

**Published:** 2015-06-17

**Authors:** Layla S Mofid, Martín Casapía, Antonio Montresor, Elham Rahme, William D Fraser, Grace S Marquis, Jozef Vercruysse, Lindsay H Allen, Theresa W Gyorkos

**Affiliations:** 1 Department of Epidemiology, Biostatistics and Occupational Health, McGill University, Montréal, Québec, Canada; 2 Research Institute of the McGill University Health Centre, Division of Clinical Epidemiology, Montréal, Québec, Canada; 3 Asociación Civil Selva Amazónica, Iquitos, Peru; 4 Department of Control of Neglected Tropical Diseases, World Health Organization, Geneva, Switzerland; 5 Département d'obstétrique et de gynécologie, Université de Sherbrooke, Sherbrooke, Québec, Canada; 6 School of Dietetics and Human Nutrition, McGill University, Ste. Anne-de-Bellevue, Québec, Canada; 7 Department of Virology, Parasitology and Immunology, Faculty of Veterinary Medicine, Ghent University, Merelbeke, Belgium; 8 USDA, ARS Western Human Nutrition Research Center, University of California, Davis, California, USA

**Keywords:** albendazole, helminthiasis, PARASITOLOGY, postpartum, randomised controlled trials, intervention study

## Abstract

**Introduction:**

Soil-transmitted helminth infections are endemic in 114 countries worldwide, and cause the highest burden of disease among all neglected tropical diseases. The WHO includes women of reproductive age as a high-risk group for infection. The primary consequence of infection in this population is anaemia. During lactation, anaemia may contribute to reduced quality and quantity of milk, decreasing the duration of exclusive breastfeeding and lowering the age at weaning. To date, no study has investigated the effects of maternal postpartum deworming on infant or maternal health outcomes.

**Methods and analysis:**

A single-centre, parallel, double-blind, randomised, placebo-controlled trial will be carried out in Iquitos, Peru, to assess the effectiveness of integrating single-dose 400 mg albendazole into routine maternal postpartum care. A total of 1010 mother-infant pairs will be randomised to either the intervention or control arm, following inhospital delivery and prior to discharge. Participants will be visited in their homes at 1, 6, 12 and 24 months following delivery for outcome ascertainment. The primary outcome is infant mean weight gain between birth and 6 months of age. Secondary outcomes include other infant growth indicators and morbidity, maternal soil-transmitted helminth infection and intensity, anaemia, fatigue, and breastfeeding practices. All statistical analyses will be performed on an intention-to-treat basis.

**Ethics and dissemination:**

Research ethics board approval has been obtained from the McGill University Health Centre (Canada), the Asociación Civil Impacta Salud y Educación (Peru) and the Instituto Nacional de Salud (Peru). A data safety and monitoring committee is in place to oversee study progression and evaluate adverse events. The results of the analyses will be published in peer-reviewed journals, and presented at national and international conferences.

**Trial registration number:**

Clinicaltrials.gov: NCT01748929.

**Strengths and limitations of this study**

- This is the first study to evaluate the effectiveness of postpartum deworming on maternal and infant health outcomes.
- Results of this trial will provide empirical evidence to inform the WHO recommendation for deworming in women of reproductive age.
- A large sample size (n=1010) allows for high-powered primary and secondary analyses.
- This is a single-centre trial which can affect generalisability of the results to other areas with different prevalence profiles and transmission patterns of soil-transmitted helminth infections.
- The study is limited to soil-transmitted helminth infections and does not include the other parasitic infections.

## Introduction

### Background

Worldwide, over two billion people are infected with intestinal worms (hookworm, *Ascaris* and *Trichuris*), collectively referred to as the soil-transmitted helminths (STHs).^1^ STH infections discriminate across socioeconomic strata, such that the most vulnerable individuals, who reside in areas where adverse health, social, and economic outcomes predominate, carry the greatest disease burden. The WHO and other organisations consider women of reproductive age to be a high-risk group for STH infection primarily because of the anaemia that is caused by hookworm^2^ and *Trichuris*^3^ (whipworm) infections. This exacerbates the already increasing iron requirements of pregnancy, with iron deficiencies extending into the early lactation period.^4^ More than 50% of pregnant women, especially in developing countries, are estimated to be anaemic.^5^ During lactation, anaemia is thought to adversely affect milk production, which can decrease the duration of exclusive breastfeeding and lower the age at weaning,^6^ and ultimately impact negatively on infant growth.

Lactating women in developing countries are at risk of suffering from a shortage of dietary fat and micronutrient deficiencies due to a suboptimal diet and parasitic infections, including STHs, which compete for micronutrients, like iron.^7^ The consequences of co-occurring malnutrition and infection in a new mother may include an impact on breast milk composition^8^and subsequently, negatively affect the nutritional status of infants.^9^ STH infections can cause reduced absorption of dietary fat leading to lower energy intake and inadequate absorption of vitamin A. Reduced transfer of vitamin A into breast milk may cause insufficient vitamin A acquisition in infant liver stores, leading to vitamin A deficiency over the first 6 months of life and beyond.^10^ Although maternal iron stores do not directly affect breast milk concentrations of iron,^11^ maternal anaemia may play a role in the frequency and duration of breastfeeding due to reduced energy levels.

To date, no study has investigated the effect of providing deworming treatment to women during the early postpartum period on infant or maternal health outcomes. In effect, the WHO recommendation to specifically include lactating women within the high-risk group of women of reproductive age in deworming campaigns^12^ is based on expert opinion and not on empirical evidence. Systematic reviews on deworming have, however, been conducted on other subgroups of women in the reproductive age (eg, non-pregnant, non-lactating women; pregnant women). A 2010 systematic review on deworming in non-pregnant populations^13^ included two studies of women in the reproductive age: one trial^14^ that specifically excluded lactating women, and one observational study^15^ that included lactating women, but in which lactation status was not ascertained. Four trials to date^16–19^ have been conducted in pregnant populations, and are summarised in three systematic reviews.^20^^–^22 None of these trials used the same intervention (ie, the same anthelminthic and micronutrient combination) or had the same follow-up time frame. While data from pregnant populations are of value, given the different nature of the interface between mother and child at the time of deworming, it is unclear whether the results are generalisable to lactating women.

### Rationale

Malnutrition is the leading cause of mortality in children under 5 years of age in developing countries, with over 150 million children classified as underweight, stunted and/or wasted.^23^ Poor nutrition predisposes children to infection, leading to increased risk of mortality,^24^ higher risk of cognitive deficits, lower educational achievement and lower productivity as adults,^25^ thus perpetuating the poverty cycle into future generations. The health of mothers and young children are intimately intertwined, and the 1000-days period from conception to the age of 2 years is a crucial time in shaping the health and development of children.^25^ ^26^ It is within this critical window when interventions can have the greatest impact on future health throughout the entire lifespan.

Deworming has been shown to be one of the safest and most cost-effective interventions for reducing disease burden in endemic countries, and is the cornerstone of prevention and control measures against STH infections.^27^ There is evidence from veterinary research in ruminants that has shown that worm infections can negatively affect the quality of milk and impair production.^28^ In addition to modifying the nutritional and immunological composition of milk,^29^ worm infections also suppress energy and protein availability prior to parturition,^30^ ^31^ which can influence feeding behaviours. Administration of deworming treatment has been shown to improve milk production in cattle.^32^ ^33^

**Figure 1:**
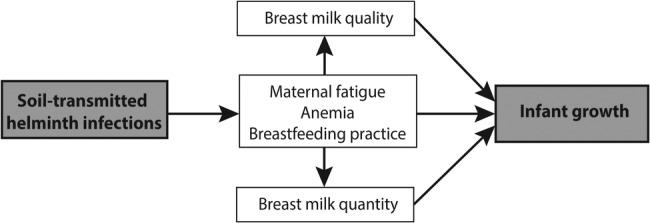
Proposed mechanism for the effect of soil-transmitted helminth infections on maternal and infant health.

The proposed deworming intervention is expected to improve capacity for breastfeeding, and quality and quantity of milk transfer to infants by reducing maternal anaemia, improving appetite and increasing energy levels. Mothers with improved nutritional status and enhanced energy levels may be more likely to initiate and continue exclusive breastfeeding to the 6-month recommended time point, and beyond. Additionally, improved maternal nutritional status may influence the passage of certain nutrients (eg, thiamin, riboflavin, vitamin B-6 and vitamin B-12, among others^34^) to breast milk during breastfeeding. This may, in turn, improve nutritional status of infants, and enhance their growth and development (figure 1). Integration of deworming into early postpartum care, therefore, has the potential to improve health outcomes for two vulnerable populations simultaneously.

The aim of the current study is to provide rigorous empirical evidence on the benefits and underlying biological mechanisms of maternal postpartum deworming in STH-endemic areas in order to inform global public health policy.

### Research objectives

#### Primary research objective

- To determine the effectiveness of maternal postpartum deworming on mean weight gain in infants between birth and six months of age.

#### Secondary research objectives

1. To determine the effectiveness of maternal postpartum deworming on the following infant outcomes: (A) weight and height indices (ie, z-scores for weight-for-age, weight-for-height and height-for-age); and (B) infant morbidity (ie, occurrence of diarrhoea, respiratory illness, fever, and ear infection in the previous 2 weeks).
2. To determine the effectiveness of maternal postpartum deworming on the following maternal outcomes: (A) STH infection and intensity; (B) anaemia; (C) self-reported fatigue; and (D) self-reported breastfeeding practice.
3. To determine the effectiveness of maternal postpartum deworming on indicators of breast milk quality, and quantity.

## Methods and analysis

### Study design and setting

A parallel, double-blind, randomised, placebo-controlled trial (RCT) will be used to examine the effectiveness of integrating single-dose albendazole administration into routine in-hospital maternal postpartum care on infant and maternal health outcomes, in Iquitos, Peru. Iquitos is the capital of the Department of Loreto in the Amazon Basin of northeastern Peru. Treatment allocation will take place within one centre, Hospital Iquitos ‘Cesar Garayar Garcia’. This hospital has a catchment area that includes individuals who reside in the poor and highly STH-endemic district of Belén. In 2004, prevalences of 45% for hookworm, 60% for *Ascaris* and 80% for *Trichuris* were reported in over 1000 pregnant women enrolled in a previous RCT.^17^

### Interventions

All women and children included in this trial will receive usual postpartum care (eg, examinations following delivery, nutritional counselling, infant vaccinations, etc), according to hospital standard of care and Ministry of Health guidelines. In addition, the following interventions with be compared.

#### Experimental group

A single-dose 400 mg albendazole tablet (oral deworming drug) will be given to women following delivery and prior to hospital discharge.

#### Control group

A single-dose 400 mg placebo tablet (identical to the experimental drug with respect to size, shape, colour, taste and smell) will be given to women following delivery and prior to hospital discharge.

### Eligibility criteria

#### Inclusion criteria

Women are eligible to participate in the trial if they meet the following criteria: (1) they deliver at Hospital Iquitos ‘Cesar Garayar Garcia’; and (2) they are likely to reside in Iquitos or a neighbouring area for the next 24 months.

#### Exclusion criteria

Women are ineligible to participate in the trial if: (1) they deliver twins or multiples; (2) they deliver a stillborn infant or an infant with a serious congenital medical condition; (3) they are transferred to another hospital prior to discharge; or if (4) they are unable to communicate in Spanish.

### Randomisation

Prior to onset of the recruitment period, a statistician not otherwise involved in the trial will produce the randomisation sequence using a computer-generated permuted block design with randomly varying block sizes of 6 and 8 with a 1:1 allocation ratio. Albendazole and matching placebo will be packaged according to the randomisation sequence into opaque, sequentially-numbered envelopes by a pharmacist and a clinician not otherwise involved in the trial, and stored in a secure temperature-controlled pharmacy.

### Outcomes

#### Primary outcome

The primary outcome is infant mean weight gain between birth and 6 months of age. Infant weight gain is thought to be the most accurate measure of breastfeeding adequacy and sufficient milk transfer from mother to breastfeeding infant.^6^ It is also an important indicator of infant growth in the first year of life.^35^ ^36^

#### Secondary outcomes

Secondary infant outcomes include weight and height indices (ie, z-scores for weight-for-age, weight-for-height and height-for-age) and WHO/UNICEF Integrated Management of Childhood Illness (IMCI) indicators of infant morbidity (ie, occurrence of diarrhoea, respiratory illness, fever, and ear infection) in the previous 2 weeks.^37^ Maternal outcomes will include the prevalence and intensity of STH infection (combined and by species), anaemia, self-reported fatigue and breastfeeding practice, and breast milk quality and quantity. All secondary outcomes will be ascertained at 1, 6, 12 and 24 months following delivery.

### Sample size and power calculations

The sample size is calculated based on the primary outcome, infant mean weight gain between birth and 6 months of age. An estimate of mean weight gain was obtained from data on children aged between 5 and 7 months residing in Belén in 2010. The estimate of 4.24 kg with a SD of 1.01 kg is the weight gain expected in the placebo group. Previous authors have claimed that an infant mean weight gain difference of approximately 500 g is clinically meaningful from trials on enriched formula feeding in infants.^36^ ^38^ ^39^ However, since the intervention for the proposed trial is given to women rather than infants, and since there is the possibility for effect dilution (ie, treating both STH infected and non-infected mothers), a mean weight gain difference of at least 200 g is expected between the two intervention groups. This difference in infant mean weight gain is also considered to be clinically significant.^40^

The sample size calculation is, therefore, based on an expected effect size of 0.2 kg and a SD of 1.01 kg, a significance level (α) of 0.05 and a power (1-β) of 0.80. As informed by a previous study in the same hospital population,^41^ the final sample size takes into account a 20% loss to follow-up. Based on the above specifications and a two-sided independent t test, we estimate that 1010 participants is the total sample size needed to declare that infant weight gain is different between intervention groups.

This trial will have sufficient power to detect a difference in proportions as low as 4% for moderate/heavy STH intensity (control group: 6% vs experimental group: 2%),^17^ as well as differences in species-specific STH infection (ie, hookworm, *Ascaris*, *Trichuris*) and infant morbidity indicators (ie, recent occurrence of diarrhoea, respiratory illness, fever, and ear infection), since the differences in proportions between intervention groups for these outcomes are expected to be even larger.^17^ ^42–44^ The study will also have sufficient power to detect a clinically meaningful mean difference of 0.5 in z-scores for weight-for-age, weight-for-height and height-for-age,^45^ and a difference in maternal haemoglobin levels similar to those observed in other studies on anthelmintic administration.^13^ ^17^ ^46^ Sample size and power calculations were conducted using PS Power and Sample Size Calculations V.3.0 (Copyright 1997 by Dupont and Plummet).

### Recruitment

Enumeration of pregnant women in their second and third trimester of pregnancy living in the study area was carried out in December 2013 and January of 2014 using rosters of pregnant women obtained from surrounding health centres and through door-to-door canvassing, in conjunction with the Regional Ministry of Health activities. A two-stage approach will be used for recruiting women into the study prior to delivery.

**Figure 2:**
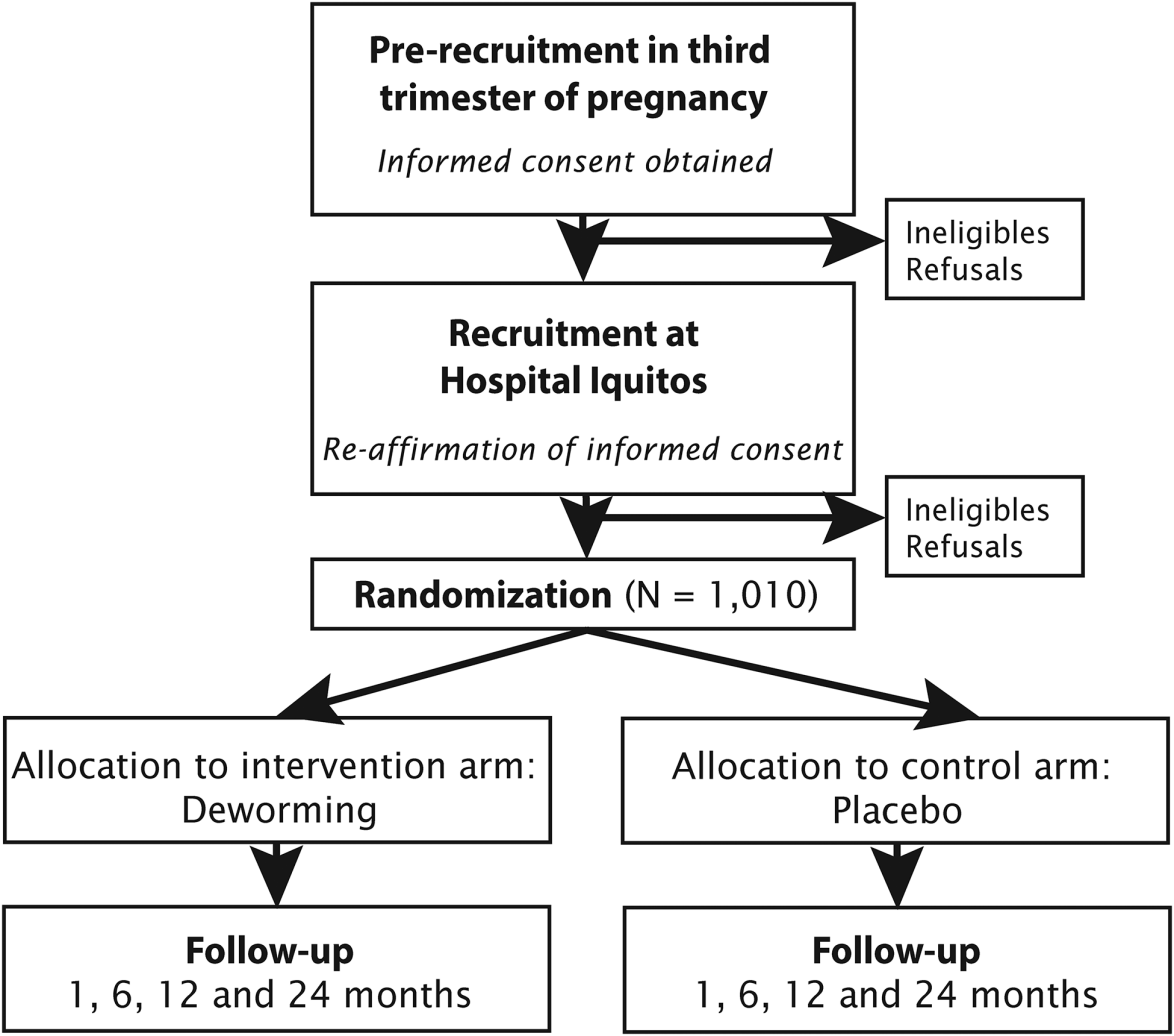
Flow diagram of the proposed randomised-controlled trial, including recruitment and follow-up. Duration of follow-up is 24 months.

In stage one, research assistants will visit the homes of women in their third trimester of pregnancy to explain the research study, obtain informed consent from both women and their partners, and administer the baseline questionnaire. Women who are interested in participating in the study will be assessed for eligibility. Informed consent will be sought at this time (figure 2).

In stage two, women presenting for delivery in the labour room of Hospital Iquitos ‘Cesar Garayar Garcia’ will be approached by a research assistant, reminded of the study, and asked whether they are still interested in taking part. Research assistants will work rotating shifts, such that at least one research assistant will be present in the labour room at all times to recruit vaginal and caesarean-section deliveries (ie, in-hospital data collection will be ongoing 24 h/day).

### Baseline assessments

Following informed consent procedures, research assistants will administer a standardised questionnaire to participants to obtain baseline information, including socio-demographics (eg, age, residence), obstetric and medical history (eg, parity, pregnancy complications), intended breastfeeding practices (eg, expected duration, exclusivity) and environmental exposures (eg, water source).

Following delivery, research assistants will extract birth date from hospital registries, including APGAR scores, gestational age, delivery type and presence of complications.

### Treatment allocation

Mother-infant pairs are normally discharged from the hospital within 24 h of a vaginal delivery and within 72 h of a caesarean-section. Research assistants on duty will bring the sequentially numbered treatment envelopes, containing the single tablet of the randomly allocated intervention, to the maternity ward. Once women have their final medical examination and receive their hospital discharge papers, they will be visited by a research assistant at their bedside and receive the next numbered treatment envelope. The research assistant will directly observe ingestion of the tablet. Participants, research assistants, outcome assessors, data analysts, and coinvestigators will be blinded to the treatment allocation.

#### Outcome ascertainment

All mother-infant pairs will be visited in their home at the specified follow-up time points by a research assistant to ascertain primary and secondary outcomes (figure 2). Data will be recorded on an application using mobile tablets, where collection and entry occurs in real time with automated question jump patterns, validation and prompts to research assistants.

#### Anthropometric measurements

Weight will be measured in duplicate at birth, 1, 6, 12 and 24 months using a portable electronic scale, accurate to the nearest 0.01 kg, calibrated daily using standard weights (Seca 354, Seca Corp., Baltimore, USA). Length will be measured in duplicate at birth, 1, 6, 12 and 24 months as recumbent crown-heel length on a flat surface using a measuring mat (Seca 417, Seca Corp., Baltimore, USA), accurate to the nearest millimetre. Both weight and length are measured in the unclothed infant. Head circumference (HC) will be measured in duplicate at birth, 1, 6, 12 and 24 months using a non-stretch Teflon measuring tape (Seca 212, Seca Corp., Baltimore, USA), accurate to the nearest millimetre. Mid-upper arm circumference (MUAC) will be measured in duplicate at 6, 12 and 24 months using a non-stretch measuring tape (UNICEF S0145620), accurate to the nearest millimetre. The mean of the first and second anthropometric measurements will be used in the analyses.

#### Infant morbidity

Research assistants will administer a questionnaire to the mothers at the 1, 6, 12 and 24 month study visits. The first component of the questionnaire is comprised of morbidity indicators modified from the IMCI Chart.^37^ Mothers will be asked about episodes of diarrhoea, fever, cough and ear infection experienced by their infant in the previous 2 weeks. These indicators have been previously used for research purposes in developing countries.^17^ ^43^ ^47^

#### Breastfeeding practice and maternal energy levels

The second component of the questionnaire is designed to assess current breastfeeding practices (eg, duration of exclusive breastfeeding, timing of first introduction of complementary foods) and maternal fatigue. Questions on breastfeeding practice have been adapted from the WHO indicators for assessing infant and young child feeding practices.^48^ Maternal fatigue is measured using the Multidimensional Assessment of Fatigue (MAF Basia Belza 1993, All rights reserved)^49^ and the Fatigue Assessment Scale (FAS).^50^

#### Maternal haemoglobin

Maternal haemoglobin levels will be measured by research assistants at the 1, 6, 12 and 24-month home visits using a HemoCue machine, accurate to within 1.5% of the gold standard reference.^51^ Blood for this test will be drawn from women by finger-prick using disposable lancets.

#### Maternal STH infection and intensity

Stool specimens will be obtained from women at baseline and at 6 months of follow-up in order to assess the prevalence and intensity of STH infections. In the case that a woman is unable to provide a stool specimen at the time of the visit, a small plastic container labelled with her unique study identification code will be left with her and a research assistant will return the following day for collection. Stool specimens will be transferred to the laboratory where these will be analysed using the Kato-Katz method by a trained microscopist. This technique is recommended for the assessment of STH prevalence and also to quantify the intensity of infection (ie, eggs per gram of stool).^52^

### Substudy

During initial informed consent procedures, women will be asked if they would like to take part in a substudy to assess breast milk quality and quantity. Of those who agree to participate, a random sample of 200 mother-infant pairs will be selected. Breast milk quality and quantity will be assessed in the subsample at 1 and 6 months following delivery. Each time point will consist of six home visits over a 2-week period, on days 1, 2, 4, 5, 14 and 15.

#### Breast milk quality assessments

**Figure 3:**
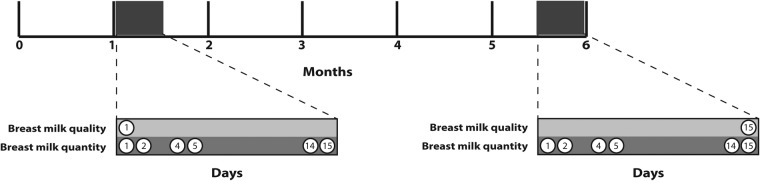
Timeline of data collection for milk quality and quantity in random sub-sample of participants. Each milk assessment is conducted over a 2-week period.

Quality of breast milk will be assessed by collecting a 50 mL milk sample from mothers. To standardise breast milk collection methods, every effort will be made to collect samples between 8:00 and noon to avoid extremes in diurnal variations.^34^ Women will be assisted in providing a breast milk sample with a hospital-grade electronic breast pump. Collection will occur by pumping milk into a presterilised tube with a leak-proof seal from the breast from which the infant has not fed for 2 h. All milk samples will be transferred to the local laboratory on ice. Macronutrient assessment will be completed on the same day of collection using the MIRIS Human Milk Analyser (HMA, Miris, Uppsala, Sweden). This machine analyzes the macronutrient composition of breast milk, including fat, protein, lactose, energy and dry matter from a 1–3 mL sample in 60 s. The remainder of each sample will be divided into two presterilised tubes and stored at −80°C; these will then be transported on dry ice to Ghent University (Belgium) and to the Western Human Nutrition Research Center (WHNRC) (California) for micronutrient and immunological assessment. Breast milk quality indicators include: IgA, IgG, lactoferrin, lysozymes, vitamins A, B1, B2, B3, B6, B12, C and D, calcium, copper, iron, zinc, free fatty acids, triglycerides and phospholipids. At 1 month following delivery, the breast milk sample will be obtained on day 1 of the 2-week assessment. At 6 months following delivery, the breast milk sample will be obtained on day 15 of the 2-week assessment (figure 3).

#### Breast milk quantity assessments

Quantity of breast milk transferred from the mother to the infant will be measured using the ‘dose-to-the-mother’ deuterium-oxide turnover technique.^53^ ^54^ In addition to optimising feasibility, efficiency and accuracy, this method is the most appropriate because it provides an estimation of water intake from both breast milk and non-breast milk sources.

On day 1, a baseline 3 mL urine sample will be collected from mothers and their infants. Subsequent to urine collection, mothers will receive a preweighed 10 g oral dose of deuterium oxide (99.8% purity), accurate to the nearest 0.001 g. Research assistants will return to participants’ homes to collect four urine samples from mothers on days 2, 5, 14 and 15, and five urine samples from infants on days 2, 4, 5, 14 and 15 (figure 3). Mothers will be asked to provide a urine sample in a small plastic container during the home visit. Infant urine samples will be obtained using paediatric urine bags. Samples will be transferred into 3 mL cryogenic tubes and stored at –20°C. Frozen urine samples will be transported to the Instituto de Nutrición y Tecnología de los Alimentos (INTA) at the University of Chile for laboratory analysis using isotope ratio mass spectrometry.

Infant water intake from breast milk and non-breast milk will be calculated by fitting isotope data to a model for water turnover in mothers and infants, and transfer of milk from mother to infant using assumptions and equations described in previous studies.^53^ ^55^ ^56^ Breast milk intake and total water intake will be expressed as g/day. This measure will represent the mean grams of breast milk transferred from mother to infant per day during each of the two 14-day assessment periods.

### Statistical analyses

#### Descriptive analyses

Study flow, including participant consent and confirmation, recruitment, eligibility, and losses to follow-up will be depicted in a Consolidated Standards of Reporting Trials (CONSORT) flow diagram. Continuous and categorical variables at baseline will be expressed as means (with SDs) and proportions, as appropriate, for description of the study population and to allow for comparisons in prognostic variables between intervention groups. Primary and secondary analyses will be performed according to the intention-to-treat principle.

#### Primary analysis

The primary outcome, infant weight gain (in grams), will be compared between the two intervention groups using a Student's t test. In addition to estimation of crude intervention effects, multivariable linear regression analysis will be used to compare infant mean weight gain between intervention groups, while adjusting for baseline covariates that were a priori determined to be important confounders in the published literature.^57^ These include maternal age, parity, deworming in the past 6 months, intention to breastfeed, and infant gestational age.^58^ Adjusted analyses will be reported as mean differences and 95% CIs.

#### Secondary analyses

Continuous outcomes will be modelled using multivariable linear regression and dichotomous outcomes will be modelled using multivariable logistic and log-linear regression methods. Log-linear regression will be used instead of logistic regression when the prevalence of the outcome is high (ie, >20%), as it is more appropriate for this situation. Secondary analyses will be adjusted for the baseline covariates mentioned above.

Sex-specific weight-for-age, weight-for-height, height-for-age, MUAC-for-age and HC-for-age z-scores will be calculated using WHO Anthro software (V.3.2.2, 2011) and macros.^59^ Underweight, wasting and stunting will be defined as z-scores for weight-for-age, weight-for-height, height-for-age, respectively of <2 SDs from the median of the WHO reference population, according to the WHO Child Growth Standards.^60^

#### Subgroup analyses

To evaluate the presence of potential effect measure modification due to infant sex, birthweight, and birth length on deworming, interaction terms will be included in separate multivariable linear regression models with the primary outcome, infant mean weight gain. These analyses will examine if there appears to be effect measure modification by sex, birthweight and birth length, and if so, they will estimate the added value of the intervention in potentially vulnerable subgroups. They will also provide insight into whether deworming can improve growth trajectories separately for boys and girls, as well as for infants born with low birthweight and birth length. If effect measure modification is found, results will be reported separately by subgroup.

## Dissemination

### Trial registration

The trial is registered with clinicaltrials.gov (NCT01748929).

### Information and informed consent

Prior to the onset of the study, meetings at health centres and the Hospital were held to inform the staff of the purpose of the trial, anticipated benefits and risks of participation, and study activities.

Written informed consent documents for participation in the trial were reviewed by the Research Ethics Boards of the McGill University Health Centre in Canada, and the Asociación Civil Impacta Salud y Educación and the Instituto Nacional de Salud in Peru. This document is written in Spanish at a basic literary level and describes the study with respect to its aims and expected contributions, the extent of participant involvement, potential benefits and inconveniences of participation, confidentiality, and the voluntary nature of initial and continued participation. During the informed consent procedure, research assistants will read the consent document to eligible women and their partners. At this time, women and their partners will have the opportunity to ask questions about the trial. Those who wish to participate in the study will be asked to provide consent by signature or fingerprint. For mothers/fathers under the age of 18 years, assent will be obtained and informed consent will be requested from their parent, guardian or spouse/partner over the age of 18 years. The Instituto Nacional de Salud in Peru requires consent from both mothers and fathers for infant participation in RCTs. Women who do not have a partner or whose partner is absent for an indefinite period of time (eg, works outside of Iquitos) will be asked to sign another document to declare the father's absence, in accordance with Peruvian ethics guidelines. Following informed consent, research assistants will administer a short 10-question evaluation to the mothers and their partners to confirm their comprehension of the document. If the mother/father responds incorrectly to a statement, the research assistant will reinforce the contents of the informed consent document.

### Trial oversight

The Trial Steering Committee consists of the principal investigator and all coinvestigators. This committee will review the study protocol, ensure the trial is being conducted in accordance with the principles of good clinical practice, and appoint three international experts with expertise in deworming, biostatistics, or clinical trial methodology to comprise the Data Safety and Monitoring Committee (DSMC). The DSMC will act in accordance with internationally recognised guidelines, review the protocol prior to study initiation, and evaluate study conduct and the occurrence of adverse events at specific time points throughout the trial (ie, after 50% and 100% of recruitment, and after the completion of each follow-up visit).

### Dissemination of results and data access

The results of this trial will be published in peer-reviewed journals, presented at various national and international fora, and communicated to international agencies, such as the WHO, who have taken a leadership role in developing global health policies pertaining to deworming in high-risk population groups. Following completion of the study, project data will initially be used by the research team to prepare the manuscripts and other standard scientific dissemination products. The final trial data set will be available for consultation and available via direct requests to the principal investigator (TWG).
